# Regional and periodic asymmetries in the effect of Russia-Ukraine war on global stock markets

**DOI:** 10.1016/j.heliyon.2024.e28362

**Published:** 2024-03-22

**Authors:** Anand Kumar Mishra, Yasmeen Ansari, Rohit Bansal, Prince Kumar Maurya

**Affiliations:** a Department of Management Studies, Rajiv Gandhi Institute of Petroleum Technology, Amethi, Uttar Pradesh, India; b Saudi Electronic University, Ministry of Higher Education, Jeddah, Saudi Arabia

**Keywords:** Event-study, Stock market, Russia-Ukraine war, Geopolitical risk, Economic policy uncertainty

## Abstract

This study aims to investigate regional and periodic asymmetries in the impact of the outbreak of the Russia-Ukraine war on global equity markets. Employing the event study methodology, the current study examines global stock market reactions within a 61-day window centred around the event day, i.e., February 24, 2022. MSCI equity indices of 47 sample countries have been utilized to ensure uniformity in the index development methodology. They provide broader coverage of global equity markets by including large and mid-cap companies, representing approximately 85% of the free float-adjusted market capitalization for each sampled country. The study extends the event window to 61 days to assess the enduring effects of the war over a relatively longer period. The research delineates regional and periodic asymmetries and posits that the impact of the war on a market is contingent upon its geographical proximity and trade relations with Russia and Ukraine. Additionally, the impact is stronger during a shorter window surrounding the event date but diminishes over the extended period.

## 1 Introduction

The outbreak of war poses a significant threat to global foreign trade relations. The most severely impacted nations being those directly involved in the conflict, followed by closely connected markets and those in close geographical proximity [1,2]. Numerous border and political conflicts have arisen worldwide, occurring even before the full recovery of global economies from the repercussions of the Covid-19 pandemic. Recent global conflicts include the clash between Indian and Chinese forces in the Galwan Valley, the Taliban's control in Afghanistan, the Armenia-Azerbaijan conflict, the military coup in Myanmar, and the border tension between China and Taiwan. One of the most recent conflicts is the intermittent Russia-Ukraine conflict, which has persisted since the 2014 annexation of Crimea but intensified due to Ukraine's expressed desire to join NATO. In response to Ukraine's NATO membership aspirations, Russia declared a special military operation in Ukraine on February 24, 2022. Since then, Ukrainian and Russian forces have been engaged in war, resulting in a significant loss of life on both sides and triggering one of the largest refugee crises in Europe since World War II. The war has led to the displacement of close to 8 million people from Ukraine. The impact on global economies varies across different conflicts, as some wars do not significantly affect the global economic landscape. However, the Russia-Ukraine war has substantial global repercussions for several reasons-

1. Russia and Ukraine collectively hold a significant stake in the global commodity market, contributing, for instance, approximately 25% of the global demand for wheat and 15% for corn and fertilizer.
2. Russia stands as the world's leading exporter of natural gas, playing a pivotal role in meeting the majority of energy requirements for European nations.
3. Following the imposition of economic sanctions by the USA on Russia, a majority of western countries have also declared substantial sanctions in response.

The imposition of such heavy sanctions, coupled with ensuing shortages of essential commodities such as food, oil, and gas, has resulted in soaring inflation globally [[3], [4], [5]]. Beyond the pronounced alterations in foreign trade dynamics, these developments have also had a significant impact on the global stock market [6]. Within the research community, there is a mounting concern, particularly regarding the far-reaching consequences of the war from economic and financial market perspectives.

The present study systematically assesses the abnormal fluctuations in the global stock market index attributed to the Russia-Ukraine war, employing a 61-day event window. The investigation delineates regional and periodic asymmetries in the impact of war on global stock markets, proposing that nations with geographic proximity and robust trade relations with Russia and Ukraine experienced more pronounced effects. Moreover, it observes that the impact was more substantial within a shorter window surrounding the event date, gradually diminishing over the extended event window.

The subsequent sections of this article are structured as follows: The second section, *‘Literature Review and Theoretical Background,’* provides an in-depth exploration of the existing knowledge base and theoretical underpinnings related to the present topic. Following this, the third section, *‘Data and Methodology,’* elucidates the dataset's characteristics and outlines the analytical procedures employed in the study. The fourth section, titled *‘Results and Discussion,’* critically deliberates on the outcomes derived from the data analysis. Subsequently, the article presents the *‘Practical Implications’* of the findings in the fifth section. Finally, the last section, *‘Conclusion and Limitations,’* encapsulates the article by offering concluding remarks and acknowledging constraints inherent in the study.

## 2 Literature Review and Theoretical Background

The Efficient Market Hypothesis posits that stock markets promptly react to unexpected events, with share prices adjusting immediately in response to new information [7]. According to this hypothesis, rational investors engage in a comprehensive evaluation of available possibilities and associated consequences, leveraging the information at their disposal to make well-informed decisions. The implication is that all pertinent data and information are swiftly incorporated into stock prices. In contrast, behavioral investment theories argue that unforeseen events trigger a rapid and sometimes irrational response from investors [8]. Furthermore, behavioral heuristics propose that individuals are inclined to give more weight to recent and easily recallable news rather than considering a comprehensive set of information [9].

The inherent heterogeneity among various frequency traders, including investors, central banks, and speculators, gives rise to differences in expectations, risk profiles, information sets, and beliefs. This diversity manifests in varied responses to identical news within the same financial market. Acknowledging this foundational premise, an appropriate analytical approach involves considering the direction and size of return and volatility connectedness within a financial system. Essentially, higher interdependence in a financial market system results in increased interconnectedness in volatility and returns [10]. The interconnectedness among global markets is not an inherent occurrence but is subject to external influences [11]. Returns and volatility within financial markets are contingent upon price oscillations influenced by events such as financial crises, misalignment of macroeconomic policies, economic policy uncertainty (EPU), geopolitical risk (GPR), oil shocks, and risks associated with speculators [12,13]. EPU and GPR emerge as pivotal indicators of uncertainty, exerting a profound influence on the connectedness of financial markets, asset pricing, and investment decisions [14,15]. EPU manifests through fluctuations in economic, monetary, fiscal, or regulatory policies, governing investors' decision-making processes [16]. It proves detrimental to economic growth and exerts adverse effects on investment, employment, and stock prices.

Asset prices respond to information concerning both deliberate and unforeseen policy adjustments on a global scale. Market participants defer their investment decisions, opting to retain liquidity as a precautionary measure against potential risks arising from prevailing uncertainty [16]. Investors also recalibrate their portfolios by inclining towards safe-haven assets, such as foreign exchange, precious metals, sustainable investments, cryptocurrencies, commodities, sovereign bonds, etc., to mitigate portfolio risk [[17], [18], [19]]. This underscores the observed "flight-to-quality" or "flight-to-safety" phenomenon, which tends to increase (reduce) the prices of risky (safe-haven) assets [14].

Similarly, the escalation of GPR, resulting from events such as wars, political tensions, coups d'état, territorial disputes, energy crises, and nuclear threats between nations, intensifies overall uncertainty [15]. It disrupts the supply chain of essential commodities for the smooth flow of the production process, leading to increased price volatility for underlying products. Volatility and return transmission across financial markets are triggered by global geopolitical uncertainty, influencing investment decisions as investors defer their choices, awaiting a more discernible economic environment during extreme events. The detrimental impact of global geopolitical risk heightens investor sentiments, prompting a shift from higher-risk to lower-risk markets [5,20]. GPR plays a pivotal role in shaping investment decisions within financial markets, as highlighted by Caldara & Iacoviello, (2022). Global financial or geopolitical bursts are not usually limited to a specific region, leading to strengthened risk transmissions among global financial markets, particularly in the short term [10,21,22].

Previously, the impact of major wars, including WWII, the Israel-Palestine War, and the Iraq War, on stock markets has been extensively examined in the American and European contexts [1,[23], [24], [25], [26], [27], [28]]. Recent research has also delved into the effects of the India-China war on sectoral returns [29]. Using the event study technique, much of the research consistently suggests that wars significantly affect stock market performance in both the short and long runs, with investors displaying panic buying and selling behavior driven by herd impulses [30]. In the context of the Russia-Ukraine conflict, several studies have explored similar themes. Boubaker et al. (2022) utilized a 7-day pre- and post-event day window and observed a heterogeneous negative impact of the war on global equity indices. Kumari et al. (2023) examined the adverse event day impact of the Russia-Ukraine war announcement on European Union stock markets. They observed varied impacts among EU countries in the post-event period, dependent on geographical proximity and market efficiency. Yousaf et al. (2022) similarly noted a negative effect on G20 market equity indices, using a 5-day pre- and post-event window [31]. Sun et al. (2022) focused on a 3-day post-event window, analyzing country-wise and sectoral equity performance. They found that countries and sectors closely situated to the war zone and deeply connected in trade relations experienced higher negative abnormal returns. The tertiary sector was more adversely affected than the manufacturing sector. Chortane & Pandey, (2022) examined the asymmetrical effect of the war on global currencies, using a 7-day post-event and a 3-day pre-event window [32]. They observed significant depreciation in European currencies, value appreciation in Pacific currencies, while those in the MEA region did not respond sharply to the occurrence of war. Maurya et al. (2023) investigated the impact of the Russia-Ukraine war on global inflation. Their findings indicated that inflation pressure increased in sample countries based on their geographical proximity and trade relations with the conflicting nations, with severe inflation observed in the majority of European economies. Kick & Rottmann, (2022) observed a positive influence of the war on abnormal returns of sustainable stocks in pre- and post-event windows [33]. However, they cautioned against using 'ESG-Hedge' during similar situations due to possible underlying factors influencing firm resilience in extreme events. Abbassi et al. (2022) conducted an event study with an 8-day post-event and 5-day pre-event window on 531 firms comprising leading equity indices of G7 nations [34]. Their observations suggested that firms with greater risk exposure and trade dependence are likely to experience negative war-induced abnormal returns. The study also considered firm-specific variables, including book-to-market ratio, firm size, financial leverage, return on assets, past returns, etc. Boungou & Yatié, (2022) performed an event study on a sample of 94 countries and observed that countries in geographical proximity to Ukraine and Russia experienced higher negative impacts. Furthermore, they presented evidence indicating that countries condemning the invasion experienced a more pronounced negative impact compared to neutral ones. The repercussions of the war on energy, tourism, and the low-carbon technology sector have also been evaluated by several researchers [[35], [36], [37], [38]].

The comprehensive review of the event study literature concerning the Russia-Ukraine war has been pivotal in identifying existing gaps. Many studies have utilized shorter event windows, despite the observed effects of the particular event persisting for a more extended period. Additionally, prior studies have employed representative indices for countries based on a non-uniform methodology with varying weights for free float-adjusted market capitalization. These studies often considered indices comprising only large-cap companies. To address these gaps, our study makes a valuable contribution by conducting an event study encompassing a total of 47 economies grouped into different categories based on similar characteristics. The use of MSCI indices for all sample countries and the benchmark index ensures uniformity in the methodology for index calculation. These indices comprehensively include both large- and mid-cap companies, representing over 85% of free float-adjusted market capitalization. Notably, our study employs a larger event window of 61 days, covering 30 days pre- and post-event, to capture the prolonged effects of the event over an extended period.

Well in advance of the war announcement, there were reports from various news sources about a substantial mobilization of Russian armed forces along the Ukrainian border. This development prompted speculations regarding the potential outbreak of war between the two countries. Consequently, the current study adopts an anticipation window of 30 days preceding the event date. Moreover, the war triggered a cascade of global reactions that were intricately linked to the war, representing a series of events stemming from its occurrence. The announcement of sanctions against Russia and the resulting disruptions in the supply chain contributed to a global increase in the prices of food products and oil supplies. The ripple effects of the war were evident worldwide, driven by geopolitical risk and economic policy uncertainty, and these effects extended over a longer time frame. Consequently, the current study incorporates a longer adjustment window of 30 days post the event day. Existing research indicates that a more extended event window provides a comprehensive understanding of the effects of an event, particularly when it involves a chain of reactions [39,40].

## 3 Data and Methodology

The sample for this study comprises 47 countries, categorized into developed and emerging economies based on the widely accepted classification by MSCI. Specifically, the developed market category includes 23 countries, while the emerging market category encompasses 24 countries. To provide a more nuanced analysis, the overall sample is further classified into distinct regional categories, including the Americas, EMEA (Europe, the Middle East, and Africa), Asia, and the Pacific. Uniformity in the methodology for index calculation is ensured by using MSCI indices for all the sample countries. Serving as the benchmark market index is the MSCI All Country World Index (ACWI), which is based on 2900 constituents and represents large- and mid-cap companies across 23 developed and 24 emerging markets. This index covers approximately 85% of the globally available equity investment opportunities.

### 3.1 Event

**Fig. 1:**
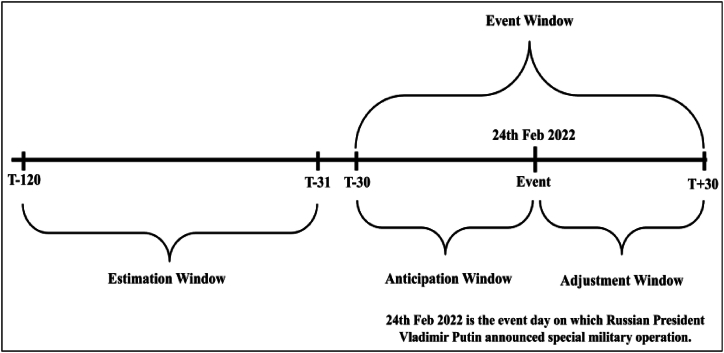
Diagrammatic presentation of estimation window and event window.

The event date, event window, estimation window, anticipation window, and adjustment window are reflected in the below diagram Fig. 1.

### 3.2 Estimation procedure

Actual return

(1) $$R_{it}=\mathrm{LN}\left(\frac{P_{it}}{P_{it-1}}\right)\times 100$$

Where,

LN is the log of natural numbers.

$P_{it}$ is the price of index i on day t; and.

$P_{it-1}$ is the price of index i on day before day t.

#### 3.2.1 Expected return/Normal return

OLS market model has been used to calculate the normal daily returns as under:

(2) $$ER_{it}=\alpha +{\beta R}_{mt}$$

Where,

$ER_{it}$ is the normal return;

**α** is the intercept and **β** is the slope of the OLS regression model calculated for the estimation window data; and.

$R_{mt}$ is the rate of return on the market benchmark index (ACWI) on day t.

#### 3.2.2 Abnormal return

(3) $$AR_{it}=R_{it}-{ER}_{mt}$$

Where,

$AR_{it}$ is the abnormal return on index i on day t;

${ER}_{mt}$ is the normal return on the index i on day t.

$R_{it}$ is the actual return on index i on day t;

#### 3.2.3 Cumulative abnormal return

(4) $${CAR}_{i}\left(p,q\right)=\sum_{t=p}^{q}{AR}_{it}$$

Where,

${CAR}_{i}$ represents CAR of the index i over day p to day q.

${AR}_{it}$ is the abnormal return calculated in the previous step.

#### 3.2.4 Average abnormal return

(5) $${AAR}_{t}=\frac{1}{N}\sum_{i=1}^{n}{AR}_{it}$$

Where,

${AAR}_{t}$ is the Average Abnormal Return on day t, and.

N is the number of indices in the group.

These AARs are then aggregated to obtain the cumulative average abnormal return (CAARs) for the event window.

### 3.3 Calculation of test statistics

#### 3.3.1 T-stat for AAR (parametric test)

(6) $${AAR}_{t}\;t=\frac{{AAR}_{t}}{\sigma_{N,e}}$$

Where.

${AAR}_{t}\;<i>t</i>$ is the T-stat of AAR

$\sigma_{N,e}$ is the aggregate estimation period standard deviation which is calculated as-

(7) $$\sigma_{N,e}=\sqrt{\frac{\sum_{i=1}^{N}\sigma_{i,e}^{2}}{N^{2}}}$$

Where,

N is the number of indices in the particular group.

$\sigma_{i,e}$ is the standard deviation of daily returns during the estimation period, which is calculated as-

(8) $$\sigma_{i,e}=\sqrt{\frac{\sum_{T-120}^{T-31}{\left(AR_{it}-{AAR}_{e}\right)}^{2}}{n}}$$

Where,

${AAR}_{e}$ is the average abnormal return of the index i for the estimation period, and.

n is the number of days in the estimation period.

T-stat for CAAR

(9) $${CAAR}_{t}\;t=\frac{{CAAR}_{t}}{\sigma_{N,e}\sqrt{N_{t+1}}}$$

Where,

${CAAR}_{t}$ is the CAAR on day t, and.

$N_{t+1}$ is the number of days for which ${AAR}_{t}$ is cumulated plus 1.

### 3.4 Robustness check

#### 3.4.1 Corrado test statistic (non-parametric test)

To enhance the robustness of the results, T-statistics have been supplemented with the Corrado test, which is derived from the rank of the abnormal return. The Corrado test statistics have been computed using the simplified equation proposed by Ref. [41] as follows:

(10) $${\text{Corrado}}_{t}\;\mathrm{AAR}=\sqrt{\frac{3}{N\left(T^{2}-1\right)}}\sum_{i=1}^{n}\left[2K\left({\text{AR}}_{\text{it}}\right)-\left(T-1\right)\right]$$

Where,

**N** is the number of indices in the group.

**T** is the number of abnormal returns used in the study i.e., for 151 days.

$K\left({\text{AR}}_{\text{it}}\right)$ is the rank of the abnormal return of the i index over 151 days.

## 4 Results and Discussion

The Russian invasion of Ukraine has spurred uncertainty, leading to significant upheaval in global financial markets. This study delineates the impact of the war on global stock indices using a sample of 47 countries, employing a 61-day event window. It highlights anticipated stock market behavior before the war and tracks the evolution of global stock market reactions post the war announcement. The study provides short-term result similar to several studies conducted previously [6,42,43,44]. However, the evolution of stock market reactions in the longer event window has not been studied earlier. Therefore, this study contributes novel insights into the evolving nature of global stock market responses over time subsequent to the event day.

Table 1 and Table 2 present country-wise Cumulative Abnormal Returns (CAR) and their significance tests for the 61-day event window and shorter event windows. CARs for the 61-day event window and shorter periods offer insights into the evolution of returns since the event day. Among the 47 countries in the sample, statistically significant event-day abnormal returns were observed for 20 of the 23 developed economies and 14 of the 24 emerging market economies. Notably, the USA and Portugal exhibited statistically significant positive event-day abnormal returns, while Columbia and Kuwait had insignificant positive returns. Conversely, negative event-day abnormal returns were observed for the remaining 43 countries, with 32 of them posting statistically significant negative abnormal returns. This suggests that war announcements with a higher potential for supply-chain disruptions tend to generate negative sentiment among global investors, leading to abrupt stock market declines.

**Table 1:** CAR (developed economies).

Developed Market														
Country	(T0)		(T, T+7)		(T-7, T+7)		(T, T+15)		(T-15, T+15)		(T, T+30)		(T-30, T+30)	
	AR	T-Stat	CAR	T-Stat	CAR	T-Stat	CAR	T-Stat	CAR	T-Stat	CAR	T-Stat	CAR	T-Stat
**Canada**	−0.50%	−0.903	6.75%	4.595^a^	−2.14%	−0.995	3.90%	1.813***	6.18%	1.998**	2.69%	0.885	6.74%	1.555
**USA**	2.38%	7.648^a^	1.16%	1.407	−4.81%	−3.989^a^	0.09%	0.078	0.87%	0.504	0.63%	0.371	1.78%	0.731
**Austria**	−10.66%	−8.121^a^	−19.16%	−5.519^a^	6.69%	1.316	−6.97%	−1.371	−22.80%	−3.120^a^	−15.80%	−2.198**	−33.03%	−3.223^a^
**Belgium**	−3.31%	−3.978^a^	−7.59%	−3.448^a^	2.74%	0.849	2.51%	0.779	1.00%	0.216	−0.18%	−0.040	−3.32%	−0.511
**Denmark**	−0.91%	−0.772	6.41%	2.056**	−36.46%	−7.982^a^	9.38%	2.054**	7.54%	1.149	12.31%	1.906***	14.85%	1.612
**Finland**	−4.99%	−5.775^a^	−7.10%	−3.103^a^	−9.03%	−2.696^a^	2.82%	0.842	−2.21%	−0.458	2.42%	0.511	−2.84%	−0.420
**France**	−5.03%	−6.687^a^	−7.70%	−3.865^a^	8.21%	2.816^a^	−0.70%	−0.241	−4.47%	−1.067	−5.81%	−1.410	−9.49%	−1.614
**Germany**	−5.51%	−7.317^a^	−8.92%	−4.480^a^	−11.80%	−4.049^a^	0.24%	0.081	−5.24%	−1.250	−4.92%	−1.195	−9.94%	−1.692***
**Ireland**	−6.54%	−7.454^a^	−8.29%	−3.568^a^	−10.75%	−3.161^a^	0.04%	0.012	−5.16%	−1.057	−10.77%	−2.240**	−15.74%	−2.295**
**Israel**	−0.04%	−0.054	0.53%	0.287	−14.49%	−5.388^a^	−3.00%	−1.115	−0.38%	−0.099	1.30%	0.342	−1.71%	−0.315
**Italy**	−5.51%	−6.819^a^	−10.82%	−5.055^a^	−14.06%	−4.490^a^	−6.16%	−1.966**	−10.58%	−2.349**	−7.68%	−1.735***	−10.86%	−1.719***
**Netherlands**	−3.34%	−2.925^a^	−3.91%	−1.294	0.46%	0.103	0.61%	0.138	0.07%	0.011	−1.87%	−0.299	−2.67%	−0.299
**Norway**	−2.26%	−1.890***	5.13%	1.620	−14.55%	−3.137^a^	3.68%	0.794	7.82%	1.173	5.41%	0.824	11.42%	1.220
**Portugal**	1.84%	1.681***	7.57%	2.611^a^	−4.80%	−1.132	7.53%	1.776***	4.11%	0.674	13.07%	2.179**	10.72%	1.253
**Spain**	−4.32%	−4.535^a^	−5.98%	−2.373**	5.84%	1.583	1.51%	0.408	0.17%	0.032	0.70%	0.134	1.97%	0.265
**Sweden**	−4.69%	−5.267^a^	−3.26%	−1.381	10.37%	3.004^a^	5.89%	1.708***	−0.82%	−0.165	2.19%	0.448	−6.00%	−0.862
**Switzerland**	−3.35%	−5.014^a^	−1.64%	−0.928	−8.48%	−3.280^a^	1.28%	0.495	−2.27%	−0.610	2.85%	0.780	−2.14%	−0.411
**UK**	−5.56%	−7.440^a^	−1.98%	−1.002	−7.98%	−2.761^a^	−0.75%	−0.258	−3.27%	−0.785	−0.28%	−0.068	−1.26%	−0.216
**Australia**	−4.44%	−4.374^a^	6.39%	2.376**	−2.96%	−0.751	6.55%	1.665***	11.23%	1.984**	10.32%	1.853***	11.02%	1.389
**Hongkong**	−2.19%	−2.694^a^	−4.20%	−1.954***	−5.30%	−1.682***	−2.14%	−0.681	2.61%	0.576	−1.80%	−0.405	6.09%	0.960
**Japan**	−1.61%	−1.648***	−2.21%	−0.858	5.80%	1.536	0.40%	0.105	−2.65%	−0.488	−3.09%	−0.578	−8.32%	−1.093
**New Zealand**	−5.54%	−4.980^a^	7.17%	2.439**	−6.35%	−1.474	8.97%	2.083**	8.19%	1.323	6.82%	1.120	0.25%	0.028
**Singapore**	−3.93%	−5.450^a^	−8.10%	−4.247^a^	−4.81%	−1.723***	−2.46%	−0.880	0.25%	0.062	−1.21%	−0.306	0.56%	0.099

a P-value <0.01; **P-value <0.05; ***P-value <0.10.

**Table 2:** CAR (emerging economies).

Emerging Market														
Country	Event Day(T0)		(T, T+7)		(T-7, T+7)		(T, T+15)		(T-15, T+15)		(T, T+30)		(T-30, T+30)	
	AR	T-Stat	CAR	T-Stat	CAR	T-Stat	CAR	T-Stat	CAR	T-Stat	CAR	T-Stat	CAR	T-Stat
**Brazil**	−1.58%	−0.985	7.44%	1.757***	7.52%	1.213	3.86%	0.623	17.56%	1.971**	16.32%	1.862***	47.17%	3.774*
**Chile**	−0.78%	−0.337	10.46%	1.710***	−11.34%	−1.266	9.96%	1.113	17.13%	1.331	13.27%	1.048	27.51%	1.523
**Colombia**	0.04%	0.032	7.06%	1.931***	12.87%	2.405**	4.00%	0.747	8.64%	1.123	12.10%	1.599	23.43%	2.171**
**Mexico**	−1.47%	−1.483	1.75%	0.669	10.98%	2.869*	5.10%	1.332	7.49%	1.362	7.66%	1.414	6.62%	0.858
**Peru**	−1.97%	−1.199	9.40%	2.162**	12.61%	1.981**	6.70%	1.053	17.01%	1.860***	−1.42%	−0.158	3.69%	0.288
**Czech Republic**	−9.04%	−8.270*	−14.40%	−4.975*	0.97%	0.229	−0.68%	−0.161	−9.20%	−1.510	2.42%	0.404	−8.60%	−1.007
**Egypt**	−4.96%	−4.565*	−7.32%	−2.543**	9.44%	2.241**	−7.61%	−1.809***	−17.40%	−2.875*	−20.45%	−3.435*	−35.41%	−4.170*
**Greece**	−7.48%	−7.825*	−9.01%	−3.561*	−24.05%	−6.492*	−5.74%	−1.549	−6.46%	−1.212	−5.18%	−0.990	−2.52%	−0.337
**Hungary**	−16.93%	−10.997*	−19.94%	−4.894*	−13.24%	−2.220**	1.06%	0.177	−25.62%	−2.989*	−12.28%	−1.456	−39.87%	−3.316*
**Kuwait**	0.15%	0.245	4.19%	2.625*	−13.16%	−5.628*	3.98%	1.701***	5.94%	1.766***	6.35%	1.921***	8.91%	1.890***
**Poland**	−16.14%	−13.251*	−1.28%	−0.399	−44.61%	−9.454*	14.31%	3.034*	−8.57%	−1.263	10.08%	1.510	−18.62%	−1.957***
**Qatar**	−0.80%	−1.226	7.43%	4.299*	6.56%	2.592*	4.96%	1.960**	2.00%	0.549	9.02%	2.521**	8.56%	1.678***
**Saudi Arabia**	−1.68%	−1.721***	5.18%	2.004**	−22.29%	−5.896*	2.80%	0.741	3.85%	0.709	6.13%	1.147	8.57%	1.125
**South Africa**	−2.45%	−1.792***	3.31%	0.915	5.78%	1.091	5.81%	1.098	11.73%	1.541	2.55%	0.340	10.36%	0.970
**Turkey**	−7.82%	−2.024**	14.47%	1.415	6.11%	0.408	8.66%	0.578	18.96%	0.881	15.13%	0.715	30.95%	1.025
**UAE**	−0.20%	−0.172	8.43%	2.759*	3.34%	0.746	5.01%	1.119	9.08%	1.411	8.91%	1.408	17.34%	1.922***
**China**	−2.29%	−1.903***	−4.64%	−1.457	15.53%	3.332*	−8.58%	−1.841***	−5.21%	−0.778	−7.40%	−1.124	−5.22%	−0.555
**India**	−6.03%	−6.933*	−1.12%	−0.486	9.26%	2.750*	4.65%	1.380	−1.48%	−0.305	6.03%	1.266	−2.27%	−0.335
**Indonesia**	−1.33%	−1.316	1.09%	0.407	−6.42%	−1.641	1.12%	0.286	5.10%	0.907	−1.14%	−0.206	1.98%	0.251
**Korea**	−2.97%	−2.820*	−0.57%	−0.206	−2.49%	−0.611	0.18%	0.045	4.20%	0.715	−1.68%	−0.290	−5.62%	−0.682
**Malaysia**	−1.17%	−1.622	0.95%	0.498	2.74%	0.978	1.55%	0.552	6.14%	1.527	2.14%	0.540	4.48%	0.794
**Philippines**	−2.57%	−2.320**	−0.33%	−0.114	−1.43%	−0.334	−3.42%	−0.798	−5.62%	−0.912	−5.62%	−0.928	−3.63%	−0.420
**Taiwan**	−3.02%	−3.460*	−3.28%	−1.420	0.65%	0.192	−4.90%	−1.448	−5.65%	−1.162	−9.68%	−2.023**	−14.24%	−2.086**
**Thailand**	−2.03%	−2.438**	−1.06%	−0.481	−1.66%	−0.515	−1.75%	−0.540	5.70%	1.227	−4.15%	−0.909	6.49%	0.997

P-value <0.01; **P-value <0.05; ***P-value <0.10.

For the period from the event day plus 7 days onward, CAR was significant in 15 developed economies and 12 emerging markets. Similarly, CAR for 15 days around the event day [T-7 to T+7] was significant in 14 developed economies and 12 emerging markets. Seven developed economies and five emerging markets displayed significant CAR for the event day plus 15 days onward. Similarly, four developed economies and five emerging markets exhibited significant CAR for 31 days around the event day [T-15 to T+15]. Six developed economies and five emerging market countries experienced significant CAR for the event day plus 30 days onward. Furthermore, four developed economies and nine emerging markets demonstrated significant CAR for 61 days around the event day [T-30 to T+30]. These findings underscore that a large number of countries were affected by the war in the short term around the event date. However, the impact of the war persisted even for a longer period in some countries [45]. This outcome signifies investors' continuous monitoring of diplomatic efforts, peace negotiations, and any signs of escalating tensions that could impact their investment prospects.

**Fig. 2:**
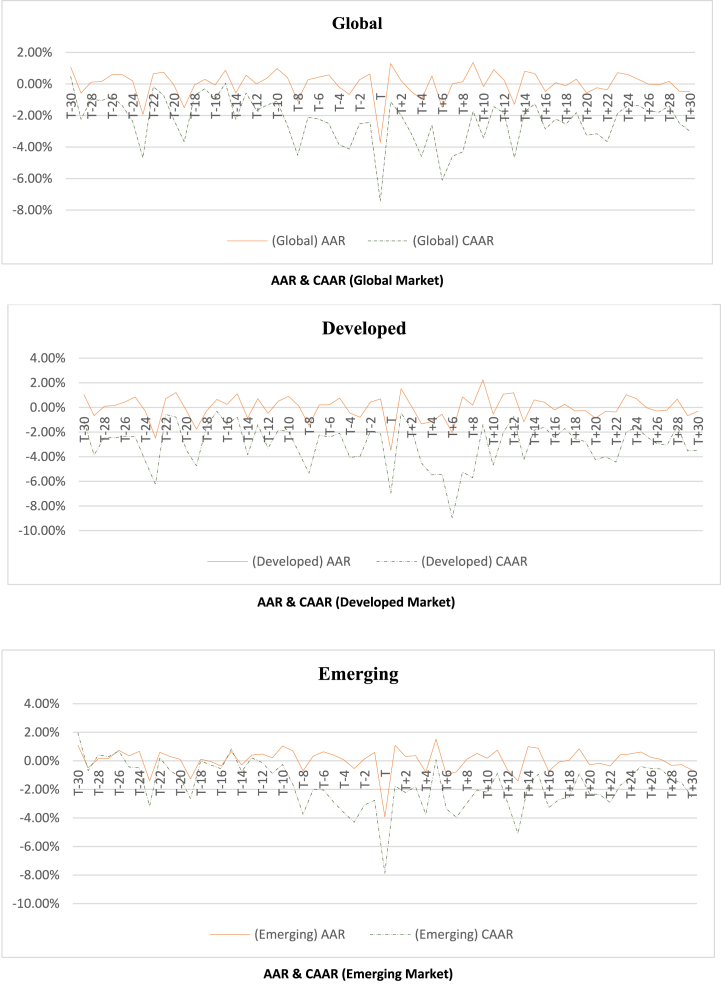

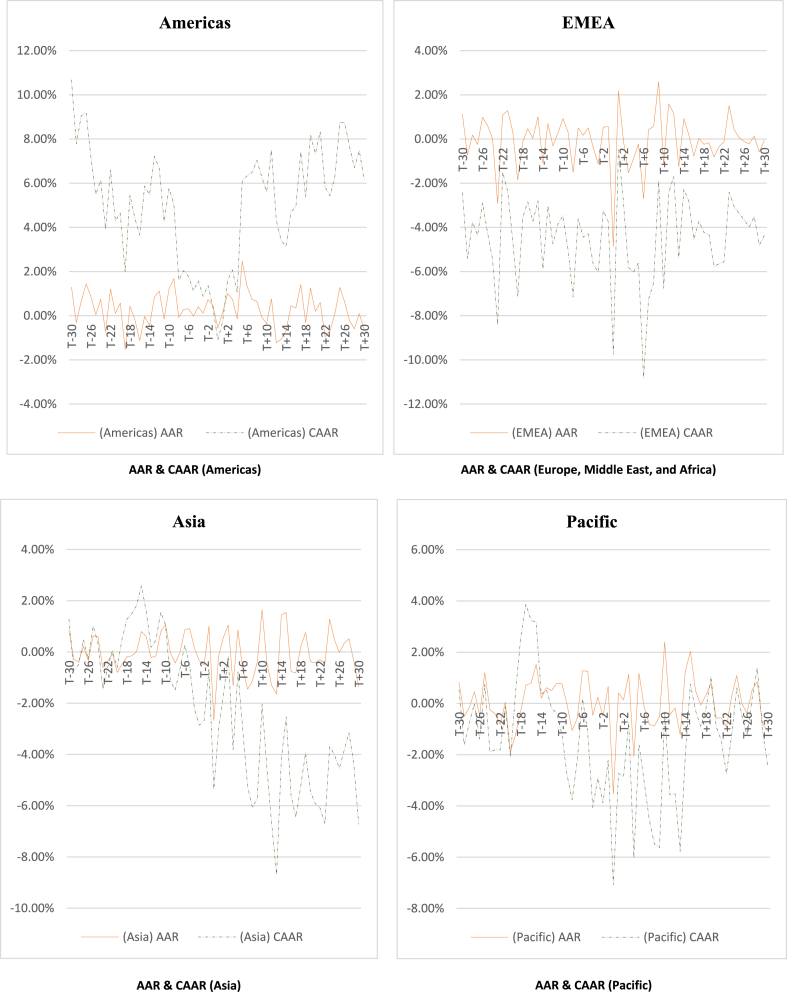
Graphical presentation of AAR and CAAR.

**Fig. 3:**
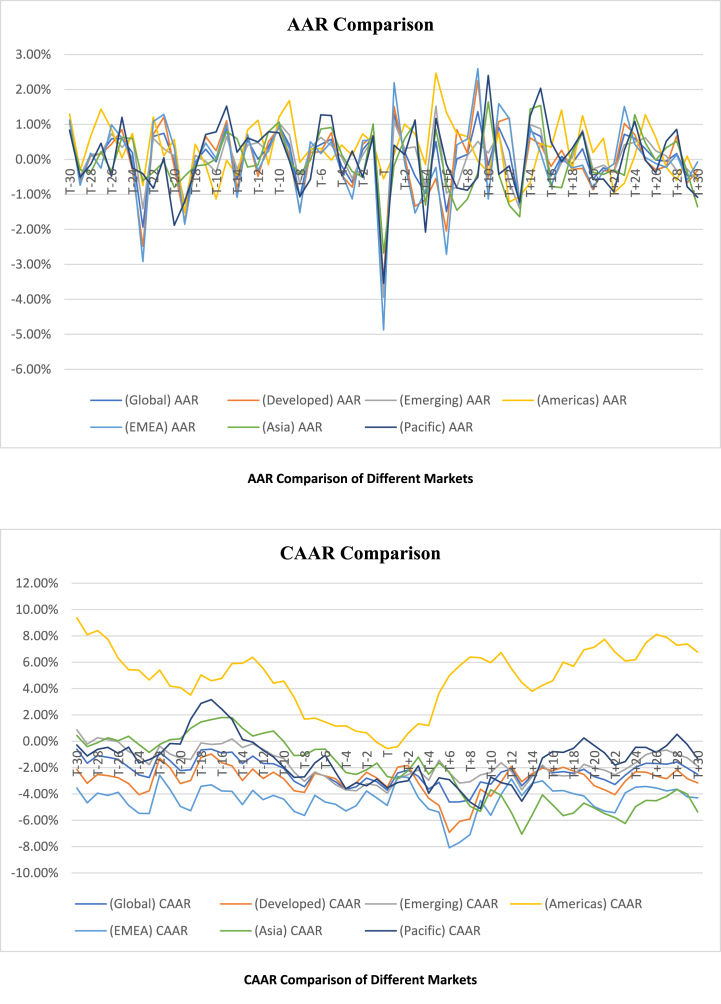
Graphical comparison of AAR and CAAR.

To analyze the regional distribution of the war's effects, the sample countries have been classified into seven groups based on MSCI classifications, namely Global, Developed, Emerging market, EMEA (Europe, the Middle East, and Africa), Americas, Pacific, and Asia. Subsequently, the study calculates the average abnormal returns (AARs) and cumulative average abnormal returns (CAARs) for these groups. The significance of AARs and CAARs is tested using T-statistics. In addition to the 61-day event window, the study examines the effects over relatively shorter window periods of 7 days and 15 days. The obtained T-stat is compared with the T-critical value, as depicted in Table 3, to assess the significance of AARs and CAARs. The computed AARs and CAARs for the different groups, along with their respective T-stats and significance, are presented in the **Appendix**. Table 4 and Table 5 provide a summary of the significant AARs and CAARs for different market groupings. Furthermore, Fig. 2 illustrates paired AARs and CAARs throughout the event window for various market classifications, while Fig. 3 visually compares AARs and CAARs for different market groups.

**Table 3:** Critical T-value.

Classification	N	d/f	Critical t-Value		
			(α = 0.01)	(α = 0.05)	(α = 0.10)
**Global**	47	46	2.69	2.01	1.68
**Developed**	23	22	2.82	2.07	1.72
**Emerging Market**	24	23	2.81	2.07	1.71
**EMEA**	27	26	2.78	2.06	1.71
**Americas**	7	6	3.71	2.45	1.94
**Pacific**	5	4	4.60	2.78	2.13
**Asia**	8	7	3.50	2.36	1.89

**Table 4:** Summary of significant AARs.

AAR																		
Market	Pre (Including event day)								Post								Total	
	10%		5%		1%		Total		10%		5%		1%		Total			
	(+)ve	(−)ve	(+)ve	(−)ve	(+)ve	(−)ve	(+)ve	(−)ve	(+)ve	(−)ve	(+)ve	(−)ve	(+)ve	(−)ve	(+)ve	(−)ve	Pre	Post
**Global**	0	0	3	0	10	7^a^	13	7	2	0	0	2	8	7	10	9	20	19
**Developed**	0	0	3	2	10	7^a^	13	9	0	1	1	1	9	7	10	9	22	19
**Emerging**	2	1	5	1	2	3^a^	9	5	1	0	2	5	5	2	8	7	14	15
**Americas**	4	1	2	1	0	0	6	2	2	2	2	0	1	0	5	2	8	7
**EMEA**	1	0	5	0	7	7^a^	13	7	2	0	1	1	6	7	9	8	20	17
**Asia**	2	1	5	0	0	1^a^	7	2	1	3	2	1	4	5	7	9	9	16
**Pacific**	0	1	4	2	0	1^a^	4	4	2	2	2	1	2	1	6	4	8	10

**Table 5:** Summary of significant CAARs.

CAAR																		
**Market**	**Pre**								**Post**								**Total**	
	**10%**		**5%**		**1%**		**Total**		**10%**		**5%**		**1%**		**Total**			
	**(+)ve**	**(−)ve**	**(+)ve**	**(−)ve**	**(+)ve**	**(−)ve**	**(+)ve**	**(−)ve**	**(+)ve**	**(−)ve**	**(+)ve**	**(−)ve**	**(+)ve**	**(−)ve**	**(+)ve**	**(−)ve**	**Pre**	**Post**
**Global**	0	1	0	3	0	6	0	10	0	0	0	3	0	8	0	11	10	11
**Developed**	0	1	0	4	0	4	0	9	0	1	0	4	0	6	0	11	9	11
**Emerging**	0	2	0	1	0	5	0	8	0	3	0	3	0	2	0	8	8	8
**Americas**	8	0	0	0	0	0	8	0	10	0	6	0	0	0	16	0	8	16
**EMEA**	0	4	0	6	0	9	0	19	0	5	0	2	0	8	0	15	19	15
**Asia**	0	1	0	3	0	1	0	5	0	7	0	12	0	3	0	22	5	22
**Pacific**	0	1	0	3	0	1	0	5	0	2	0	4	0	0	0	6	5	6

As depicted in Table 4, the number of significant Average Abnormal Returns (AARs) in the anticipation and adjustment windows was nearly equal for the global, developed, emerging, Americas, EMEA, and Pacific markets. However, in the Asian market, significant AARs in the adjustment window outnumbered those in the anticipation window. Specifically, the Americas, Asia, and Pacific regions had fewer than ten significant AARs, while twenty or more AARs were deemed significant for the global, developed, and EMEA markets in the anticipation window. This implies that EMEA countries were more adept at anticipating the war compared to the Americas, Asia, and Pacific markets. Further analysis reveals fifteen significant AARs in the adjustment window for global, developed, emerging, EMEA, and Asian markets. The Americas and Pacific markets displayed a limited number of significant AARs in both the anticipation and adjustment windows. This outcome suggests that, with the exception of the Americas and Pacific, all other markets were significantly impacted by the war. In the anticipation window, the number of significant positive AARs exceeded negative AARs for all market classifications except the Pacific, which exhibited an equal number of positive and negative AARs. Similarly, in the adjustment window, the number of significant positive AARs exceeded negative AARs for all market classifications except Asia, where a greater number of negative AARs than positive AARs were observed. This suggests that the Asian market faced greater challenges in the adjustment window than other groups, possibly due to a lag in anticipating the war and its potential repercussions in the pre-event period. In contrast, other markets demonstrated better anticipation of the war before its occurrence compared to the Asian market.

As indicated in Table 5, EMEA exhibited nineteen significantly negative Cumulative Average Abnormal Returns (CAARs) in the anticipation window, surpassing the ten or fewer observed in all other market classifications. This underscores that EMEA was the region most profoundly affected by the war during the anticipation period. Further, more than ten negative CAARs were recorded for global, developed, EMEA, and Asian markets in the adjustment window. In contrast, emerging markets and Pacific markets displayed fewer than ten significant CAARs. Interestingly, no significant positive CAARs were observed in the anticipation window or the adjustment window for any market classification except the Americas. The Americas recorded eight significant positive CAARs in the anticipation window and sixteen in the adjustment window. Notably, the Americas did not experience a significant negative CAAR in either the anticipation window or the adjustment window.

In summary, these findings suggest that the war had a substantial and predominantly negative impact on the EMEA region during the anticipation period. While various markets, including global, developed, EMEA, and Asian markets, experienced notable negative CAARs in the adjustment window, emerging markets and Pacific markets displayed fewer significant CAARs. The Americas, on the other hand, demonstrated a unique response with significant positive CAARs in both the anticipation and adjustment windows, signifying significant benefits and outperformance compared to other market classifications.

### 4.1 Robustness check

The parametric T-test has been complemented with the Corrado test to ensure the robustness of the results. Table 6 and Table 7 provide a comparison of the T-statistic and Corrado-test values for the event window. Both the test statistics for almost all AARs produced similar results at their respective significant levels, ensuring the robustness of the outcomes.

**Table 6:** Robustness test (pre-event period).

Days	Global		Developed		Emerging		Americas		EMEA		Asia		Pacific	
	T-Stat	C-Value	T-Stat	C-Value	T-Stat	C-Value	T-Stat	C-Value	T-Stat	C-Value	T-Stat	C-Value	T-Stat	C-Value
**T-30**	6.13	6.857	5.51	4.76	3.75	4.94	2.42	2.12	4.70	5.625	2.47	3.12	1.96	1.67
**T-29**	−3.29	−3.059	−3.52	−2.45	−1.66	−1.88	−0.58	0.16	−3.09	−3.338	−0.86	−0.56	−1.20	−0.71
**T-28**	0.69	0.371	0.49	0.68	0.51	−0.15	1.23	0.85	0.75	0.857	−1.10	−1.20	−0.36	−0.51
**T-27**	0.92	0.813	0.81	0.90	0.57	0.25	2.70	1.82	−1.03	−0.675	0.65	0.92	1.10	1.35
**T-26**	3.38	3.236	2.29	2.70	2.55	1.88	1.62	1.52	4.16	4.252	−0.99	−1.47	−1.14	−0.32
**T-25**	3.40	3.694	4.47	4.15	1.19	1.11	0.08	−0.52	2.56	3.457	1.84	1.38	2.86	2.32
**T-24**	1.12	0.187	−1.53	−1.53	2.30	1.76	1.39	−0.01	0.07	−0.411	1.74	1.70	−0.58	−0.41
**T-23**	−11.10	−7.057	−13.02	−5.82	−4.88	−4.18	−1.39	−1.41	−12.26	−7.347	−1.77	−1.44	−0.97	−1.07
**T-22**	3.76	3.450	3.75	2.89	2.07	1.99	2.28	2.23	4.56	4.940	−1.06	−1.06	−1.96	−2.18
**T-21**	4.30	3.858	6.35	4.64	1.06	0.86	0.20	−0.32	5.40	5.143	−0.19	0.02	0.11	0.06
**T-20**	−0.33	−0.050	−1.20	−0.26	0.37	0.18	1.08	1.24	1.41	1.320	−2.32	−1.05	−4.47	−2.88
**T-19**	−8.62	−6.913	−9.19	−5.33	−4.37	−4.46	−2.86	−2.76	−7.79	−6.336	−1.39	−0.97	−2.86	−1.26
**T-18**	−0.38	−0.810	−1.35	−1.21	0.41	0.05	0.81	1.12	−0.50	−0.892	−0.54	−0.30	−0.69	−0.63
**T-17**	1.67	2.540	3.42	3.31	−0.19	0.31	−0.32	−0.03	1.96	3.121	−0.45	−0.27	1.69	1.13
**T-16**	−0.38	−0.606	1.31	0.30	−1.27	−1.14	−2.11	−2.75	0.12	0.177	0.03	0.54	1.87	0.85
**T-15**	5.02	4.738	5.80	4.27	2.25	2.45	−0.05	−0.46	4.24	4.247	2.32	2.24	3.62	2.36
**T-14**	−3.25	−3.222	−4.54	−3.55	−0.96	−1.03	−0.85	−0.86	−4.55	−4.751	1.73	1.67	0.50	0.36
**T-13**	3.20	3.798	3.74	3.71	1.42	1.68	1.58	1.66	2.96	3.510	−0.64	−0.11	1.46	1.76
**T-12**	0.06	−0.505	−2.52	−1.94	1.67	1.19	2.10	1.34	−1.33	−1.669	−0.46	−0.45	1.19	1.35
**T-11**	2.05	2.543	2.66	2.24	0.74	1.37	−0.28	−0.82	1.18	1.422	2.30	2.67	1.88	2.10
**T-10**	5.60	5.756	4.76	4.33	3.61	3.81	2.26	1.41	3.89	4.358	3.12	3.20	1.81	1.93
**T-9**	2.39	2.306	0.65	0.87	2.41	2.38	3.15	2.85	1.28	1.179	−0.09	0.60	−0.09	0.31
**T-8**	−6.15	−5.448	−7.64	−5.25	−2.43	−2.48	−0.15	−0.15	−6.39	−6.084	−1.27	−0.70	−2.52	−1.65
**T-7**	1.54	2.175	1.07	1.55	1.15	1.53	0.54	0.50	2.12	3.015	−0.06	0.24	−1.36	−1.25
**T-6**	2.45	2.607	1.14	0.44	2.18	3.21	0.59	0.20	0.71	1.117	2.53	2.97	3.03	2.29
**T-5**	3.30	3.396	4.00	3.54	1.38	1.28	−0.04	−0.28	2.12	2.168	2.66	2.43	2.99	2.60
**T-4**	−1.09	−1.429	−2.39	−2.23	0.23	0.19	0.77	0.85	−1.64	−2.159	0.40	0.89	−1.10	−1.43
**T-3**	−3.81	−3.504	−4.16	−2.90	−1.87	−2.06	0.22	0.75	−4.74	−4.671	−1.01	−0.52	0.57	0.71
**T-2**	1.59	1.784	2.26	2.43	0.45	0.12	1.37	1.61	2.26	3.033	-1.48	−1.49	−1.20	−1.09
**T-1**	3.65	4.605	3.61	3.84	2.03	2.68	0.87	0.27	2.36	3.541	2.96	3.03	1.59	1.75
**T**	−21.31	−8.898	−18.24	−5.97	−13.64	−6.61	−1.04	−1.40	−20.46	−6.998	−7.80	−3.95	−8.42	−3.61

**Table 7:** Robustness test (post-event period).

Days	Global		Developed		Emerging		Americas		EMEA		Asia		Pacific	
	T-Stat	C-Value	T-Stat	C-Value	T-Stat	C-Value	T-Stat	C-Value	T-Stat	C-Value	T-Stat	C-Value	T-Stat	C-Value
**T**	−21.31	−8.898	−18.24	−5.97	−13.64	−6.61	−1.04	−1.40	−20.46	−6.998	−7.80	−3.95	−8.42	−3.61
**T+1**	7.44	3.644	7.93	4.27	3.77	0.92	0.27	−0.01	9.21	4.751	−0.48	0.20	0.96	0.50
**T+2**	1.29	2.225	0.70	0.52	1.09	2.61	1.89	1.95	−0.29	0.834	1.71	2.47	0.33	0.22
**T+3**	−2.71	0.468	−7.02	−2.65	1.24	3.25	1.37	1.60	−6.43	−2.499	3.06	2.86	2.68	1.75
**T+4**	−5.55	−4.735	−6.04	−3.94	−2.74	−2.77	−0.28	−0.32	−3.67	−2.857	−3.80	−3.11	−4.93	−3.08
**T+5**	2.92	1.482	−2.88	−2.36	5.27	4.39	4.62	3.53	−0.95	−1.929	2.52	2.43	2.79	1.93
**T+6**	−8.56	−4.514	−10.73	−4.08	−3.32	−2.33	2.51	2.66	−11.38	−5.815	−2.04	−1.89	−0.33	−0.42
**T+7**	0.09	1.007	4.43	2.93	−2.69	−1.46	1.40	1.33	1.75	3.272	−4.23	−3.02	−1.94	−1.56
**T+8**	0.82	0.816	0.97	0.65	0.36	0.51	1.22	1.36	2.44	2.384	−3.31	−1.80	−2.09	−2.07
**T+9**	7.82	2.560	11.80	3.53	1.78	0.13	−0.10	−0.68	10.90	4.428	−1.12	−0.31	−1.20	−0.77
**T+10**	−0.96	−0.910	−2.80	−1.73	0.63	0.42	−0.68	−1.06	−4.75	−4.212	4.79	4.10	5.71	3.56
**T+11**	5.23	4.307	5.67	4.13	2.60	1.99	1.44	1.60	6.69	5.757	−1.25	−1.32	−1.01	−0.84
**T+12**	1.42	0.686	6.22	3.11	−2.25	−2.09	−2.28	−2.67	4.92	2.857	−3.82	−0.72	−0.44	−0.68
**T+13**	−7.40	−6.612	−6.12	−4.25	−4.87	−5.09	−1.99	−1.92	−5.28	−5.779	−4.77	−2.33	−2.91	−0.89
**T+14**	4.64	3.350	3.22	2.22	3.45	2.51	−1.25	−2.26	3.88	4.230	4.19	1.47	2.95	2.11
**T+15**	3.77	3.008	2.24	1.24	3.04	2.99	0.85	0.62	0.81	0.821	4.49	3.28	4.84	3.16
**T+16**	−2.71	−2.968	−1.05	−1.06	−2.54	−3.11	0.65	0.49	−3.25	−3.497	−2.25	−1.55	1.18	1.15
**T+17**	0.48	0.174	1.37	0.85	−0.30	−0.59	2.64	2.50	0.14	0.380	−2.35	−1.98	−0.15	−0.21
**T+18**	−0.68	−0.596	−1.50	−1.22	0.15	0.36	−0.59	−0.91	−1.01	−0.596	0.57	0.39	0.74	0.95
**T+19**	1.74	1.382	−1.33	−1.50	2.90	3.40	2.34	1.47	−0.70	−0.848	2.20	2.68	1.88	1.88
**T+20**	−3.23	−3.156	−4.53	−3.84	−0.95	−0.66	0.37	0.24	−3.42	−3.091	−1.08	−0.64	−1.36	−1.54
**T+21**	−1.36	−1.409	−1.64	−1.24	−0.57	−0.76	1.13	1.66	−1.43	−1.766	−1.24	−0.26	−1.34	−1.50
**T+22**	−2.10	−1.640	−1.94	−1.44	−1.25	−0.89	−1.80	−2.13	−0.53	0.137	−0.87	−0.43	−2.20	−1.69
**T+23**	4.12	1.780	5.40	3.03	1.46	−0.48	−1.27	−1.99	6.35	4.018	−1.32	−1.29	0.62	0.47
**T+24**	3.43	3.229	3.73	2.66	1.69	1.91	0.19	0.16	1.82	1.444	3.72	3.57	2.58	1.84
**T+25**	1.73	1.770	−0.16	−0.26	2.15	2.73	2.39	2.31	0.24	0.243	1.38	1.99	−0.03	−0.13
**T+26**	−0.10	−0.676	−1.54	−1.55	0.86	0.57	1.18	1.43	−0.49	−1.170	−0.07	0.46	−0.91	−0.91
**T+27**	−0.29	−0.569	−1.08	−0.87	0.34	0.06	−0.41	−0.75	−0.95	−1.258	0.98	1.22	1.24	1.54
**T+28**	0.97	1.971	3.53	2.73	−1.09	0.09	−1.11	−1.31	0.57	1.400	1.51	2.20	2.04	1.70
**T+29**	−2.67	−2.362	−3.51	−3.00	−0.94	−0.37	0.17	0.39	−2.46	−1.921	−1.02	−1.06	−1.85	−2.20
**T+30**	−2.74	−3.222	−1.66	−1.90	−2.20	−2.65	−1.16	−1.37	−0.30	−0.702	−3.94	−2.50	−2.57	−2.75

## 5 Practical Implications

The findings of the current study strongly indicate that the EMEA region bore the most significant negative impact of the Russia-Ukraine war, whereas the Americas benefited the most. While the war had a pronounced effect on other economies, this impact was more transient and subsided over time. These insights hold crucial implications for both policymakers and investors. For policymakers, the study underscores the importance of diversifying supply chain sources for essential commodities and energy supplies. Emphasizing and promoting renewable energy sources as integral components of the energy mix can mitigate dependence on limited suppliers with substantial fossil fuel reserves. The study suggests that overreliance on a single market for essential commodities and energy needs could be detrimental to an economy in times of adversity. In light of these implications, policymakers are encouraged to strategize and implement measures that enhance supply chain resilience and reduce vulnerability to external shocks. Diversification and sustainability should be central considerations in energy and commodity sourcing strategies. This approach can contribute to the overall economic stability and resilience of nations in the face of geopolitical uncertainties and conflicts.

The American and Pacific markets emerged as the least affected by the Russia-Ukraine war. In fact, the American market experienced positive effects in both the anticipation and adjustment windows. This positive impact can be attributed to the geographical distance of American markets from the war zone, coupled with the imposition of sanctions by various countries that opened avenues for American exports of energy supplies to Europe and neighboring regions. However, while short-term benefits were evident for the American markets due to abnormal price fluctuations, the study suggests that they experienced significant long-term negative repercussions. In particular, inflationary spirals and substantial rate increments were observed, posing challenges to sustained economic growth. To mitigate supply-chain disruptions and ensure sustainable growth, policymakers are advised to prioritize efforts towards fostering peace and stability. While investors may have benefited in the short run from abnormal price fluctuations, the study underscores the importance of monitoring diplomatic efforts, peace negotiations, and signs of escalating tensions to inform long-term investment decisions. On the other hand, Asian markets lagged in anticipating the event and were least affected during the anticipation window. However, the war had a negative impact on the Asian market in the post-event window. This can be attributed to the fact that many Asian economies are net oil importers, and the shortage of oil supplies in the market could lead to a sharp increase in oil prices, subsequently causing higher inflation and lower GDP growth. The study emphasizes the significance of proactive measures in Asian economies to anticipate and respond to such events, safeguarding against potential economic challenges in the aftermath of geopolitical conflicts.

The majority of global markets experienced a negative event-day abnormal return following the Russia-Ukraine war announcement. However, the impact of the war announcement gradually diminished over time from the event date. Specifically, for a shorter window of 7 days post-event and 15 days around the event, over 25 countries displayed significant Cumulative Abnormal Returns (CARs). This number decreased to ten countries for a longer event window of 15- and 30-days post-event and 31- and 61- days around the event day.

Short-term traders can capitalize on abnormal price fluctuations by implementing timely and appropriate trading strategies in their respective markets. For instance, traders in the European market might have benefited from holding short positions during the adjustment period, while traders in the American market could have benefited from long positions. On the other hand, long-term investors can view the price crash in their respective markets as an opportunity to average down their cost of investment, using the dip as a chance to invest a lump-sum amount with a longer time horizon.

In conclusion, geopolitical risks and economic policy uncertainty present both opportunities and challenges for different markets. Investors are advised to maintain a well-diversified portfolio across various asset classes, regions, and sectors to reduce risk [5,20]. Diversification serves as a means to mitigate the impact of localized geopolitical events on a portfolio. Staying informed about geopolitical developments and regularly monitoring diplomatic efforts, peace negotiations, and signs of escalation is crucial for informed investment decisions. Allocating a portion of the portfolio to defensive stocks, which are less sensitive to economic downturns, and considering industries such as utilities, healthcare, and consumer staples that tend to be more resilient during periods of uncertainty can be prudent. Additionally, investors should assess the geographical exposure of their portfolio and consider shifting investments towards regions that may be more stable or less directly affected by the conflict. Including safe-haven assets such as gold, government bonds, cryptocurrencies, or foreign currency can also serve as a hedge against volatility [[17], [18], [19]].

## 6 Conclusion and Limitations

The event study methodology employed in the current research, utilizing a 61-day window, sheds light on the profound impact of the Russia-Ukraine war on global equity markets. The choice of a larger event window signifies the understanding that the announcement of war is not a singular event confined to a single day. Instead, it triggers a chain of reactions, particularly when it significantly disrupts the global supply chain system. The observed worldwide reactions were directly attributable to the war announcement rather than being isolated incidents. Consequently, the equity indices of global markets were heavily influenced. Notably, the asymmetrical effect of the war was evident, depending on the proximity to the war zone and the market's level of connectedness.

The long-term ramifications of the Russia-Ukraine war have been substantial, pushing several developed and emerging economies to the brink of recession. There is a pressing need for a more in-depth understanding of how war sets off a cascade of events, creates a spiral of problems, and impacts global economies at the macro level. Future studies could delve into the effects of a critical war on various macroeconomic indicators such as inflation, interest rates, exchange rates, GDP growth, and others. It is important to acknowledge that the current study relied on MSCI indices from 47 countries, selected based on data availability, to ensure a uniform index development methodology. However, it is noted that MSCI has not yet developed benchmark indices for several countries, and therefore, the findings concerning different market groups may not precisely represent these countries. This limitation should be considered when interpreting the study's results and may offer avenues for further research and exploration.

## Data availability statement

Data associated with this study will be made available on request.

## CRediT authorship contribution statement

**Anand Kumar Mishra:** Writing – review & editing, Writing – original draft, Visualization, Validation, Supervision, Software, Resources, Project administration, Methodology, Investigation, Formal analysis, Data curation, Conceptualization. **Yasmeen Ansari:** Writing – review & editing, Visualization, Supervision, Funding acquisition. **Rohit Bansal:** Writing – review & editing, Visualization, Supervision, Project administration, Conceptualization. **Prince Kumar Maurya:** Writing – review & editing, Visualization, Validation, Supervision, Methodology.

## Declaration of competing interest

The authors declare that they have no known competing financial interests or personal relationships that could have appeared to influence the work reported in this paper.
