# Supplementary material for: Regional and periodic asymmetries in the effect of Russia-Ukraine war on global stock markets
Source: Heliyon. 2024 Mar 22;10(7):e28362. doi: 10.1016/j.heliyon.2024.e28362 (PMC10979091; doi:10.1016/j.heliyon.2024.e28362)
Supplement: Multimedia component 1 [file mmc1.docx]

**Table 1- AAR (Pre-Event Period)**

| **Days** | **Global** | | **Developed** | | **Emerging** | | **Americas** | | **EMEA** | | **Asia** | | **Pacific** | |
| --- | --- | --- | --- | --- | --- | --- | --- | --- | --- | --- | --- | --- | --- | --- |
|  | **AAR** | **T-Stat** | **AAR** | **T-Stat** | **AAR** | **T-Stat** | **AAR** | **T-Stat** | **AAR** | **T-Stat** | **AAR** | **T-Stat** | **AAR** | **T-Stat** |
| **T-30** | 1.07% | 6.13* | 1.05% | 5.51* | 1.08% | 3.75* | 1.29% | 2.42*** | 1.12% | 4.70* | 0.85% | 2.47** | 0.83% | 1.96 |
| **T-29** | -0.57% | -3.29* | -0.67% | -3.52* | -0.48% | -1.66 | -0.31% | -0.58 | -0.74% | -3.09* | -0.30% | -0.86 | -0.50% | -1.20 |
| **T-28** | 0.12% | 0.69 | 0.09% | 0.49 | 0.15% | 0.51 | 0.65% | 1.23 | 0.18% | 0.75 | -0.38% | -1.10 | -0.15% | -0.36 |
| **T-27** | 0.16% | 0.92 | 0.15% | 0.81 | 0.17% | 0.57 | 1.44% | 2.70** | -0.25% | -1.03 | 0.22% | 0.65 | 0.46% | 1.10 |
| **T-26** | 0.59% | 3.38* | 0.44% | 2.29** | 0.74% | 2.55** | 0.86% | 1.62 | 0.99% | 4.16* | -0.34% | -0.99 | -0.48% | -1.14 |
| **T-25** | 0.59% | 3.40* | 0.85% | 4.47* | 0.34% | 1.19 | 0.04% | 0.08 | 0.61% | 2.56** | 0.63% | 1.84 | 1.20% | 2.86** |
| **T-24** | 0.20% | 1.12 | -0.29% | -1.53 | 0.66% | 2.30** | 0.74% | 1.39 | 0.02% | 0.07 | 0.60% | 1.74 | -0.25% | -0.58 |
| **T-23** | -1.93% | -11.10* | -2.48% | -13.02* | -1.41% | -4.88* | -0.74% | -1.39 | -2.92% | -12.26* | -0.61% | -1.77 | -0.41% | -0.97 |
| **T-22** | 0.65% | 3.76* | 0.72% | 3.75* | 0.60% | 2.07*** | 1.21% | 2.28*** | 1.09% | 4.56* | -0.36% | -1.06 | -0.82% | -1.96 |
| **T-21** | 0.75% | 4.30* | 1.21% | 6.35* | 0.31% | 1.06 | 0.11% | 0.20 | 1.29% | 5.40* | -0.06% | -0.19 | 0.05% | 0.11 |
| **T-20** | -0.06% | -0.33 | -0.23% | -1.20 | 0.11% | 0.37 | 0.57% | 1.08 | 0.34% | 1.41 | -0.80% | -2.32*** | -1.88% | -4.47** |
| **T-19** | -1.50% | -8.62* | -1.75% | -9.19* | -1.26% | -4.37* | -1.53% | -2.86** | -1.85% | -7.79* | -0.48% | -1.39 | -1.20% | -2.86** |
| **T-18** | -0.07% | -0.38 | -0.26% | -1.35 | 0.12% | 0.41 | 0.43% | 0.81 | -0.12% | -0.50 | -0.18% | -0.54 | -0.29% | -0.69 |
| **T-17** | 0.29% | 1.67 | 0.65% | 3.42* | -0.05% | -0.19 | -0.17% | -0.32 | 0.47% | 1.96*** | -0.15% | -0.45 | 0.71% | 1.69 |
| **T-16** | -0.07% | -0.38 | 0.25% | 1.31 | -0.37% | -1.27 | -1.13% | -2.11*** | 0.03% | 0.12 | 0.01% | 0.03 | 0.79% | 1.87 |
| **T-15** | 0.87% | 5.02* | 1.11% | 5.80* | 0.65% | 2.25** | -0.03% | -0.05 | 1.01% | 4.24* | 0.80% | 2.32*** | 1.52% | 3.62** |
| **T-14** | -0.57% | -3.25* | -0.87% | -4.54* | -0.28% | -0.96 | -0.45% | -0.85 | -1.08% | -4.55* | 0.59% | 1.73 | 0.21% | 0.50 |
| **T-13** | 0.56% | 3.20* | 0.71% | 3.74* | 0.41% | 1.42 | 0.84% | 1.58 | 0.70% | 2.96* | -0.22% | -0.64 | 0.61% | 1.46 |
| **T-12** | 0.01% | 0.06 | -0.48% | -2.52** | 0.48% | 1.67 | 1.12% | 2.10*** | -0.32% | -1.33 | -0.16% | -0.46 | 0.50% | 1.19 |
| **T-11** | 0.36% | 2.05** | 0.51% | 2.66** | 0.21% | 0.74 | -0.15% | -0.28 | 0.28% | 1.18 | 0.79% | 2.30*** | 0.79% | 1.88 |
| **T-10** | 0.98% | 5.60* | 0.91% | 4.76* | 1.04% | 3.61* | 1.21% | 2.26*** | 0.93% | 3.89* | 1.07% | 3.12** | 0.76% | 1.81 |
| **T-9** | 0.42% | 2.39** | 0.12% | 0.65 | 0.70% | 2.41** | 1.68% | 3.15** | 0.31% | 1.28 | -0.03% | -0.09 | -0.04% | -0.09 |
| **T-8** | -1.07% | -6.15* | -1.46% | -7.64* | -0.70% | -2.43** | -0.08% | -0.15 | -1.52% | -6.39* | -0.44% | -1.27 | -1.06% | -2.52*** |
| **T-7** | 0.27% | 1.54 | 0.20% | 1.07 | 0.33% | 1.15 | 0.29% | 0.54 | 0.51% | 2.12** | -0.02% | -0.06 | -0.57% | -1.36 |
| **T-6** | 0.43% | 2.45** | 0.22% | 1.14 | 0.63% | 2.18** | 0.31% | 0.59 | 0.17% | 0.71 | 0.87% | 2.53** | 1.27% | 3.03** |
| **T-5** | 0.58% | 3.30* | 0.76% | 4.00* | 0.40% | 1.38 | -0.02% | -0.04 | 0.51% | 2.12** | 0.91% | 2.66** | 1.26% | 2.99** |
| **T-4** | -0.19% | -1.09 | -0.46% | -2.39** | 0.07% | 0.23 | 0.41% | 0.77 | -0.39% | -1.64 | 0.14% | 0.40 | -0.46% | -1.10 |
| **T-3** | -0.66% | -3.81* | -0.79% | -4.16* | -0.54% | -1.87*** | 0.12% | 0.22 | -1.13% | -4.74* | -0.35% | -1.01 | 0.24% | 0.57 |
| **T-2** | 0.28% | 1.59 | 0.43% | 2.26** | 0.13% | 0.45 | 0.73% | 1.37 | 0.54% | 2.26** | -0.51% | -1.48 | -0.51% | -1.20 |
| **T-1** | 0.64% | 3.65* | 0.69% | 3.61* | 0.59% | 2.03*** | 0.47% | 0.87 | 0.56% | 2.36** | 1.02% | 2.96** | 0.67% | 1.59 |
| **T** | -3.71% | -21.31* | -3.48% | -18.24* | -3.94% | -13.64* | -0.55% | -1.04 | -4.87% | -20.46* | -2.68% | -7.80* | -3.54% | -8.42* |

| **Days** | **Global** | | **Developed** | | **Emerging** | | **Americas** | | **EMEA** | | **Asia** | | **Pacific** | |
| --- | --- | --- | --- | --- | --- | --- | --- | --- | --- | --- | --- | --- | --- | --- |
|  | **AAR** | **T-Stat** | **AAR** | **T-Stat** | **AAR** | **T-Stat** | **AAR** | **T-Stat** | **AAR** | **T-Stat** | **AAR** | **T-Stat** | **AAR** | **T-Stat** |
| **T** | -3.71% | -21.31* | -3.48% | -18.24* | -3.94% | -13.64* | -0.55% | -1.04 | -4.87% | -20.46* | -2.68% | -7.80* | -3.54% | -8.42* |
| **T+1** | 1.30% | 7.44* | 1.51% | 7.93* | 1.09% | 3.77* | 0.14% | 0.27 | 2.19% | 9.21* | -0.17% | -0.48 | 0.40% | 0.96 |
| **T+2** | 0.23% | 1.29 | 0.13% | 0.70 | 0.31% | 1.09 | 1.01% | 1.89 | -0.07% | -0.29 | 0.59% | 1.71 | 0.14% | 0.33 |
| **T+3** | -0.47% | -2.71* | -1.34% | -7.02* | 0.36% | 1.24 | 0.73% | 1.37 | -1.53% | -6.43* | 1.05% | 3.06** | 1.13% | 2.68*** |
| **T+4** | -0.97% | -5.55* | -1.15% | -6.04* | -0.79% | -2.74** | -0.15% | -0.28 | -0.87% | -3.67* | -1.30% | -3.80* | -2.08% | -4.93* |
| **T+5** | 0.51% | 2.92* | -0.55% | -2.88* | 1.52% | 5.27* | 2.46% | 4.62* | -0.23% | -0.95 | 0.86% | 2.52** | 1.17% | 2.79** |
| **T+6** | -1.49% | -8.56* | -2.05% | -10.73* | -0.96% | -3.32* | 1.34% | 2.51** | -2.71% | -11.38* | -0.70% | -2.04*** | -0.14% | -0.33 |
| **T+7** | 0.02% | 0.09 | 0.84% | 4.43* | -0.78% | -2.69** | 0.74% | 1.40 | 0.42% | 1.75*** | -1.45% | -4.23* | -0.82% | -1.94 |
| **T+8** | 0.14% | 0.82 | 0.18% | 0.97 | 0.10% | 0.36 | 0.65% | 1.22 | 0.58% | 2.44** | -1.14% | -3.31** | -0.88% | -2.09 |
| **T+9** | 1.36% | 7.82* | 2.25% | 11.80* | 0.51% | 1.78*** | -0.05% | -0.10 | 2.59% | 10.90* | -0.39% | -1.12 | -0.50% | -1.20 |
| **T+10** | -0.17% | -0.96 | -0.53% | -2.80** | 0.18% | 0.63 | -0.36% | -0.68 | -1.13% | -4.75* | 1.65% | 4.79* | 2.40% | 5.71* |
| **T+11** | 0.91% | 5.23* | 1.08% | 5.67* | 0.75% | 2.60** | 0.77% | 1.44 | 1.59% | 6.69* | -0.43% | -1.25 | -0.42% | -1.01 |
| **T+12** | 0.25% | 1.42 | 1.19% | 6.22* | -0.65% | -2.25** | -1.22% | -2.28*** | 1.17% | 4.92* | -1.31% | -3.82* | -0.19% | -0.44 |
| **T+13** | -1.29% | -7.40* | -1.17% | -6.12* | -1.41% | -4.87* | -1.06% | -1.99*** | -1.26% | -5.28* | -1.64% | -4.77* | -1.23% | -2.91** |
| **T+14** | 0.81% | 4.64* | 0.61% | 3.22* | 0.99% | 3.45* | -0.67% | -1.25 | 0.92% | 3.88* | 1.44% | 4.19* | 1.24% | 2.95** |
| **T+15** | 0.66% | 3.77* | 0.43% | 2.24** | 0.88% | 3.04* | 0.45% | 0.85 | 0.19% | 0.81 | 1.54% | 4.49* | 2.03% | 4.84* |
| **T+16** | -0.47% | -2.71* | -0.20% | -1.05 | -0.73% | -2.54** | 0.35% | 0.65 | -0.77% | -3.25* | -0.77% | -2.25*** | 0.50% | 1.18 |
| **T+17** | 0.08% | 0.48 | 0.26% | 1.37 | -0.09% | -0.30 | 1.41% | 2.64** | 0.03% | 0.14 | -0.81% | -2.35*** | -0.06% | -0.15 |
| **T+18** | -0.12% | -0.68 | -0.29% | -1.50 | 0.04% | 0.15 | -0.31% | -0.59 | -0.24% | -1.01 | 0.20% | 0.57 | 0.31% | 0.74 |
| **T+19** | 0.30% | 1.74*** | -0.25% | -1.33 | 0.84% | 2.90* | 1.25% | 2.34*** | -0.17% | -0.70 | 0.76% | 2.20*** | 0.79% | 1.88 |
| **T+20** | -0.56% | -3.23* | -0.86% | -4.53* | -0.27% | -0.95 | 0.20% | 0.37 | -0.81% | -3.42* | -0.37% | -1.08 | -0.57% | -1.36 |
| **T+21** | -0.24% | -1.36 | -0.31% | -1.64 | -0.17% | -0.57 | 0.60% | 1.13 | -0.34% | -1.43 | -0.43% | -1.24 | -0.56% | -1.34 |
| **T+22** | -0.37% | -2.10** | -0.37% | -1.94*** | -0.36% | -1.25 | -0.96% | -1.80 | -0.13% | -0.53 | -0.30% | -0.87 | -0.93% | -2.20*** |
| **T+23** | 0.72% | 4.12* | 1.03% | 5.40* | 0.42% | 1.46 | -0.68% | -1.27 | 1.51% | 6.35* | -0.45% | -1.32 | 0.26% | 0.62 |
| **T+24** | 0.60% | 3.43* | 0.71% | 3.73* | 0.49% | 1.69 | 0.10% | 0.19 | 0.43% | 1.82*** | 1.28% | 3.72* | 1.09% | 2.58*** |
| **T+25** | 0.30% | 1.73*** | -0.03% | -0.16 | 0.62% | 2.15** | 1.28% | 2.39*** | 0.06% | 0.24 | 0.47% | 1.38 | -0.01% | -0.03 |
| **T+26** | -0.02% | -0.10 | -0.29% | -1.54 | 0.25% | 0.86 | 0.63% | 1.18 | -0.12% | -0.49 | -0.02% | -0.07 | -0.38% | -0.91 |
| **T+27** | -0.05% | -0.29 | -0.21% | -1.08 | 0.10% | 0.34 | -0.22% | -0.41 | -0.23% | -0.95 | 0.34% | 0.98 | 0.52% | 1.24 |
| **T+28** | 0.17% | 0.97 | 0.67% | 3.53* | -0.31% | -1.09 | -0.59% | -1.11 | 0.14% | 0.57 | 0.52% | 1.51 | 0.86% | 2.04 |
| **T+29** | -0.47% | -2.67** | -0.67% | -3.51* | -0.27% | -0.94 | 0.09% | 0.17 | -0.59% | -2.46** | -0.35% | -1.02 | -0.78% | -1.85 |
| **T+30** | -0.48% | -2.74* | -0.32% | -1.66 | -0.63% | -2.20** | -0.62% | -1.16 | -0.07% | -0.30 | -1.35% | -3.94* | -1.08% | -2.57*** |

**Table 2-AAR (Post-Event Period)**

**Table 3- CAAR- (Pre-Event Period)**

| **Days** | **Global** | | **Developed** | | **Emerging** | | **Americas** | | **EMEA** | | **Asia** | | **Pacific** | |
| --- | --- | --- | --- | --- | --- | --- | --- | --- | --- | --- | --- | --- | --- | --- |
|  | **CAAR** | **T-Stat** | **CAAR** | **T-Stat** | **CAAR** | **T-Stat** | **CAAR** | **T-Stat** | **CAAR** | **T-Stat** | **CAAR** | **T-Stat** | **CAAR** | **T-Stat** |
| **T-30** | -0.60% | -0.24 | -2.14% | -0.75 | 0.87% | 0.31 | 9.38% | 2.37*** | -3.56% | -1.11 | 0.44% | 0.16 | -0.29% | -0.09 |
| **T-29** | -1.67% | -0.68 | -3.19% | -1.14 | -0.21% | -0.08 | 8.09% | 2.08*** | -4.67% | -1.49 | -0.41% | -0.16 | -1.11% | -0.35 |
| **T-28** | -1.10% | -0.45 | -2.52% | -0.92 | 0.27% | 0.10 | 8.40% | 2.19*** | -3.94% | -1.28 | -0.11% | -0.04 | -0.61% | -0.20 |
| **T-27** | -1.22% | -0.51 | -2.61% | -0.97 | 0.12% | 0.05 | 7.74% | 2.06*** | -4.12% | -1.36 | 0.26% | 0.10 | -0.46% | -0.15 |
| **T-26** | -1.38% | -0.59 | -2.77% | -1.05 | -0.04% | -0.02 | 6.31% | 1.71 | -3.87% | -1.30 | 0.04% | 0.02 | -0.92% | -0.31 |
| **T-25** | -1.97% | -0.86 | -3.21% | -1.23 | -0.78% | -0.30 | 5.44% | 1.50 | -4.86% | -1.66 | 0.38% | 0.16 | -0.44% | -0.15 |
| **T-24** | -2.56% | -1.14 | -4.06% | -1.59 | -1.12% | -0.45 | 5.40% | 1.52 | -5.47% | -1.91*** | -0.25% | -0.11 | -1.65% | -0.57 |
| **T-23** | -2.75% | -1.25 | -3.77% | -1.51 | -1.78% | -0.73 | 4.66% | 1.34 | -5.49% | -1.96*** | -0.85% | -0.36 | -1.40% | -0.49 |
| **T-22** | -0.82% | -0.38 | -1.28% | -0.52 | -0.38% | -0.16 | 5.40% | 1.58 | -2.57% | -0.94 | -0.24% | -0.10 | -0.99% | -0.36 |
| **T-21** | -1.47% | -0.70 | -2.00% | -0.84 | -0.97% | -0.41 | 4.19% | 1.25 | -3.66% | -1.36 | 0.12% | 0.05 | -0.17% | -0.06 |
| **T-20** | -2.22% | -1.08 | -3.21% | -1.37 | -1.28% | -0.56 | 4.08% | 1.25 | -4.94% | -1.88*** | 0.19% | 0.09 | -0.21% | -0.08 |
| **T-19** | -2.17% | -1.08 | -2.98% | -1.31 | -1.39% | -0.62 | 3.50% | 1.10 | -5.28% | -2.06** | 0.99% | 0.46 | 1.67% | 0.64 |
| **T-18** | -0.66% | -0.34 | -1.23% | -0.55 | -0.12% | -0.06 | 5.03% | 1.62 | -3.43% | -1.37 | 1.46% | 0.70 | 2.87% | 1.14 |
| **T-17** | -0.60% | -0.31 | -0.97% | -0.45 | -0.24% | -0.11 | 4.60% | 1.52 | -3.31% | -1.36 | 1.65% | 0.81 | 3.16% | 1.29 |
| **T-16** | -0.89% | -0.48 | -1.62% | -0.77 | -0.19% | -0.09 | 4.77% | 1.63 | -3.77% | -1.60 | 1.80% | 0.91 | 2.45% | 1.03 |
| **T-15** | -0.82% | -0.46 | -1.87% | -0.92 | 0.18% | 0.09 | 5.90% | 2.07*** | -3.80% | -1.66 | 1.79% | 0.93 | 1.66% | 0.72 |
| **T-14** | -1.70% | -0.98 | -2.98% | -1.51 | -0.47% | -0.24 | 5.92% | 2.15*** | -4.81% | -2.17** | 0.99% | 0.53 | 0.14% | 0.06 |
| **T-13** | -1.13% | -0.68 | -2.11% | -1.11 | -0.19% | -0.10 | 6.38% | 2.40*** | -3.73% | -1.74*** | 0.40% | 0.22 | -0.07% | -0.03 |
| **T-12** | -1.69% | -1.05 | -2.83% | -1.54 | -0.60% | -0.33 | 5.53% | 2.16*** | -4.43% | -2.15** | 0.62% | 0.36 | -0.68% | -0.33 |
| **T-11** | -1.70% | -1.10 | -2.35% | -1.33 | -1.08% | -0.63 | 4.41% | 1.79 | -4.12% | -2.07** | 0.77% | 0.47 | -1.18% | -0.59 |
| **T-10** | -2.06% | -1.39 | -2.85% | -1.69 | -1.30% | -0.78 | 4.56% | 1.94*** | -4.40% | -2.31** | -0.01% | -0.01 | -1.98% | -1.03 |
| **T-9** | -3.04% | -2.14** | -3.76% | -2.33** | -2.34% | -1.48 | 3.36% | 1.49 | -5.32% | -2.94* | -1.09% | -0.72 | -2.74% | -1.50 |
| **T-8** | -3.45% | -2.57** | -3.88% | -2.54** | -3.04% | -2.02*** | 1.67% | 0.79 | -5.63% | -3.27* | -1.06% | -0.73 | -2.70% | -1.56 |
| **T-7** | -2.38% | -1.88*** | -2.43% | -1.68 | -2.33% | -1.65 | 1.75% | 0.87 | -4.11% | -2.53** | -0.62% | -0.46 | -1.64% | -1.01 |
| **T-6** | -2.65% | -2.23** | -2.63% | -1.95*** | -2.67% | -2.01*** | 1.46% | 0.78 | -4.61% | -3.04* | -0.60% | -0.47 | -1.07% | -0.70 |
| **T-5** | -3.08% | -2.80* | -2.85% | -2.28** | -3.30% | -2.69** | 1.15% | 0.66 | -4.78% | -3.41* | -1.47% | -1.25 | -2.35% | -1.66 |
| **T-4** | -3.65% | -3.64* | -3.61% | -3.17* | -3.69% | -3.30* | 1.17% | 0.74 | -5.29% | -4.13* | -2.38% | -2.22*** | -3.60% | -2.79** |
| **T-3** | -3.46% | -3.86* | -3.15% | -3.09* | -3.76% | -3.76* | 0.76% | 0.54 | -4.90% | -4.27* | -2.51% | -2.62** | -3.14% | -2.71*** |
| **T-2** | -2.80% | -3.61* | -2.36% | -2.67** | -3.22% | -3.72* | 0.64% | 0.52 | -3.77% | -3.80* | -2.17% | -2.61** | -3.38% | -3.37** |
| **T-1** | -3.08% | -4.85* | -2.79% | -3.87* | -3.35% | -4.74* | -0.09% | -0.09 | -4.31% | -5.32* | -1.66% | -2.45** | -2.87% | -3.51** |
| **T** | -3.71% | -8.29* | -3.48% | -6.82* | -3.94% | -7.87* | -0.55% | -0.78 | -4.87% | -8.50* | -2.68% | -5.58* | -3.54% | -6.12* |

**Table 4- CAAR (Post-Event Period)**

| **Days** | **Global** | | **Developed** | | **Emerging** | | **Americas** | | **EMEA** | | **Asia** | | **Pacific** | |
| --- | --- | --- | --- | --- | --- | --- | --- | --- | --- | --- | --- | --- | --- | --- |
|  | **CAAR** | **T-Stat** | **CAAR** | **T-Stat** | **CAAR** | **T-Stat** | **CAAR** | **T-Stat** | **CAAR** | **T-Stat** | **CAAR** | **T-Stat** | **CAAR** | **T-Stat** |
| **T+1** | -2.42% | -3.81* | -1.97% | -2.73** | -2.85% | -4.03* | -0.41% | -0.41 | -2.68% | -3.31* | -2.84% | -4.19* | -3.14% | -3.84** |
| **T+2** | -2.19% | -2.82* | -1.83% | -2.08** | -2.54% | -2.93* | 0.60% | 0.49 | -2.75% | -2.77** | -2.25% | -2.71** | -3.00% | -2.99** |
| **T+3** | -2.66% | -2.97* | -3.17% | -3.11* | -2.18% | -2.18** | 1.33% | 0.94 | -4.28% | -3.73* | -1.21% | -1.26 | -1.87% | -1.62 |
| **T+4** | -3.63% | -3.62* | -4.32% | -3.79* | -2.97% | -2.65** | 1.19% | 0.75 | -5.15% | -4.02* | -2.51% | -2.34*** | -3.95% | -3.05** |
| **T+5** | -3.12% | -2.84* | -4.87% | -3.90* | -1.44% | -1.18 | 3.65% | 2.10*** | -5.38% | -3.83* | -1.65% | -1.40 | -2.78% | -1.96 |
| **T+6** | -4.61% | -3.89* | -6.92% | -5.13* | -2.40% | -1.82*** | 4.99% | 2.65** | -8.09% | -5.34* | -2.35% | -1.85 | -2.92% | -1.91 |
| **T+7** | -4.60% | -3.63* | -6.08% | -4.21* | -3.18% | -2.25** | 5.73% | 2.85** | -7.67% | -4.73* | -3.80% | -2.80** | -3.73% | -2.28*** |
| **T+8** | -4.45% | -3.31* | -5.89% | -3.85* | -3.08% | -2.05*** | 6.39% | 2.99** | -7.09% | -4.13* | -4.94% | -3.43** | -4.61% | -2.66*** |
| **T+9** | -3.09% | -2.18** | -3.64% | -2.26** | -2.56% | -1.62 | 6.34% | 2.82** | -4.50% | -2.48** | -5.32% | -3.51* | -5.12% | -2.80** |
| **T+10** | -3.26% | -2.19** | -4.18% | -2.47** | -2.38% | -1.43 | 5.97% | 2.53** | -5.63% | -2.96* | -3.68% | -2.31*** | -2.72% | -1.42 |
| **T+11** | -2.35% | -1.51 | -3.09% | -1.75*** | -1.63% | -0.94 | 6.74% | 2.74** | -4.03% | -2.03*** | -4.10% | -2.47** | -3.14% | -1.57 |
| **T+12** | -2.10% | -1.30 | -1.91% | -1.04 | -2.28% | -1.26 | 5.52% | 2.15*** | -2.86% | -1.39 | -5.42% | -3.13** | -3.33% | -1.60 |
| **T+13** | -3.39% | -2.02** | -3.08% | -1.61 | -3.69% | -1.97*** | 4.46% | 1.68 | -4.12% | -1.92*** | -7.05% | -3.93* | -4.55% | -2.10 |
| **T+14** | -2.58% | -1.49 | -2.46% | -1.25 | -2.69% | -1.39 | 3.80% | 1.38 | -3.20% | -1.44 | -5.61% | -3.02** | -3.31% | -1.48 |
| **T+15** | -1.92% | -1.07 | -2.03% | -1.00 | -1.81% | -0.91 | 4.25% | 1.49 | -3.00% | -1.31 | -4.07% | -2.12*** | -1.28% | -0.55 |
| **T+16** | -2.39% | -1.29 | -2.23% | -1.06 | -2.55% | -1.23 | 4.60% | 1.57 | -3.78% | -1.60 | -4.84% | -2.45** | -0.78% | -0.33 |
| **T+17** | -2.31% | -1.21 | -1.97% | -0.91 | -2.63% | -1.24 | 6.01% | 1.99*** | -3.74% | -1.54 | -5.65% | -2.77** | -0.85% | -0.34 |
| **T+18** | -2.43% | -1.24 | -2.26% | -1.02 | -2.59% | -1.19 | 5.69% | 1.84 | -3.99% | -1.60 | -5.45% | -2.61** | -0.54% | -0.21 |
| **T+19** | -2.12% | -1.06 | -2.51% | -1.10 | -1.75% | -0.78 | 6.94% | 2.18*** | -4.15% | -1.62 | -4.70% | -2.19*** | 0.25% | 0.10 |
| **T+20** | -2.69% | -1.31 | -3.38% | -1.45 | -2.02% | -0.88 | 7.14% | 2.19*** | -4.97% | -1.89*** | -5.07% | -2.30*** | -0.32% | -0.12 |
| **T+21** | -2.92% | -1.39 | -3.69% | -1.54 | -2.19% | -0.93 | 7.74% | 2.32*** | -5.31% | -1.97*** | -5.49% | -2.44** | -0.88% | -0.32 |
| **T+22** | -3.29% | -1.53 | -4.06% | -1.66 | -2.55% | -1.06 | 6.78% | 1.99*** | -5.43% | -1.98*** | -5.79% | -2.52** | -1.81% | -0.65 |
| **T+23** | -2.57% | -1.17 | -3.03% | -1.21 | -2.13% | -0.87 | 6.10% | 1.75 | -3.92% | -1.40 | -6.24% | -2.66** | -1.54% | -0.55 |
| **T+24** | -1.97% | -0.88 | -2.32% | -0.91 | -1.64% | -0.66 | 6.20% | 1.74 | -3.49% | -1.22 | -4.97% | -2.07*** | -0.46% | -0.16 |
| **T+25** | -1.67% | -0.73 | -2.35% | -0.90 | -1.02% | -0.40 | 7.48% | 2.06*** | -3.43% | -1.17 | -4.49% | -1.84 | -0.47% | -0.16 |
| **T+26** | -1.69% | -0.72 | -2.64% | -1.00 | -0.77% | -0.30 | 8.11% | 2.20*** | -3.55% | -1.19 | -4.51% | -1.81 | -0.85% | -0.28 |
| **T+27** | -1.74% | -0.73 | -2.85% | -1.06 | -0.67% | -0.25 | 7.89% | 2.10*** | -3.77% | -1.24 | -4.18% | -1.65 | -0.33% | -0.11 |
| **T+28** | -1.57% | -0.65 | -2.18% | -0.79 | -0.99% | -0.37 | 7.30% | 1.91 | -3.64% | -1.18 | -3.66% | -1.42 | 0.53% | 0.17 |
| **T+29** | -2.04% | -0.83 | -2.85% | -1.02 | -1.26% | -0.46 | 7.39% | 1.90 | -4.22% | -1.35 | -4.01% | -1.53 | -0.25% | -0.08 |
| **T+30** | -2.51% | -1.01 | -3.16% | -1.11 | -1.89% | -0.68 | 6.77% | 1.71 | -4.29% | -1.35 | -5.36% | -2.01*** | -1.33% | -0.41 |
